# Comparison of Remimazolam-Flumazenil versus Propofol for Rigid Bronchoscopy: A Prospective Randomized Controlled Trial

**DOI:** 10.3390/jcm12010257

**Published:** 2022-12-29

**Authors:** Yafei Pan, Mo Chen, Fulei Gu, Jinyan Chen, Wen Zhang, Zhangxiang Huang, Dapeng Zhu, Jia Song, Jun Fang, Weifeng Yu, Kangjie Xie

**Affiliations:** 1 Department of Anesthesiology, Zhejiang Cancer Hospital, Research Center for Neuro-Oncology Interaction, Institute of Basic Medicine and Cancer (IBMC), Chinese Academy of Sciences, Hangzhou 310022, China; 2 Graduate School, Wannan Medical College, Wuhu 241000, China; 3 Graduate School, Wenzhou Medical University, Wenzhou 325035, China; 4 Department of Endoscopy, Zhejiang Cancer Hospital, Institute of Basic Medicine and Cancer (IBMC), Chinese Academy of Sciences, Hangzhou 310022, China

**Keywords:** remimazolam, propofol, recovery time from anesthesia, rigid bronchoscopy, flumazenil, loss of consciousness, hemodynamic fluctuations

## Abstract

Background: Remimazolam is a novel ultrashort-acting intravenous benzodiazepine sedative–hypnotic that significantly reduces the times to sedation onset and recovery. This trial was conducted to confirm the recovery time from anesthesia of remimazolam-flumazenil versus propofol in patients undergoing endotracheal surgery under rigid bronchoscopy. Methods: Patients undergoing endotracheal tumor resection or stent implantation were randomly allocated into a remimazolam group (Group R) or a propofol group (Group P). The primary outcome was the recovery time from general anesthesia. The secondary outcomes were the time to loss of consciousness (LoC), hemodynamic fluctuations, and adverse events. Results: A total of 34 patients were screened, and 30 patients were enrolled in the study. The recovery time was significantly shorter for Group R (140 ± 52 s) than for Group P (374 ± 195 s) (*p* < 0.001). The times to LoC were 76 ± 40 s in Group R and 75 ± 25 s in Group P and were not significantly different. There were also no significant differences in hemodynamic fluctuations or adverse events between the two groups. Conclusions: The recovery time from general anesthesia in rigid bronchoscopy patients was shorter using remimazolam-flumazenil than with propofol, with no dramatic hemodynamic fluctuations and adverse events or differences between the agents. Remimazolam-flumazenil allows for faster recovery from anesthesia than propofol.

## 1. Introduction

Patients with intrabronchial tumors or tracheoesophageal leaks are routinely treated using endotracheal tumor resection or stent implantation under rigid endoscopy. However, these patients usually have laborious breathing complications caused by airway stenosis or air leakage. Moreover, ventilation failure readily occurs in these patients during surgical anesthesia. In such events, it is therefore necessary to urgently restore the patient′s spontaneous breathing to reduce the threat to life caused by ventilation failure. Hence, faster recovery from anesthesia in such patients is critical.

Propofol is the most widely used anesthetic in rigid bronchoscopy but has no specific antagonist, and its elimination is mainly influenced by cardiac output and maintained hepatic perfusion. Long duration and high doses use of propofol can lead to significant recovery delay and respiratory depression [1]. Therefore, when such patients are anesthetized with propofol, faster recovery from anesthesia is limited, and patients and anesthesiologists therefore face high risks with this anesthesia. Anesthesiologists have thus been seeking general anesthetic drugs with good anesthetic effects and short recovery time from anesthesia.

Remimazolam benzenesulfonate is a novel ultrashort-acting benzodiazepine that acts on GABA receptors, increases the influx of chloride ions, leads to hyperpolarization of the nerve cell membrane, and inhibits neuronal activity, thus generating sedation and anesthesia [2]. It is characterized as facilitating rapid onset and fast offset of sedation and anesthesia [3,4] and exhibits minimal hemodynamic instability [5,6,7], even in elderly patients [8]. In addition, remimazolam is hydrolyzed into an inactive metabolite by tissue esterase independently of liver or kidney function, and it has a context-sensitive half-life of 7.5 min, similar to remifentanil [9,10,11]. It can also be antagonized by flumazenil [12,13]. In addition, it was reported that the efficacy of remimazolam was noninferior to propofol as a sedative-hypnotic [14]. In summary, remimazolam has a short metabolism time and a specific antagonist.

It is unclear, thus far, the recovery time of remimazolam-flumazenil in rigid bronchoscopy procedures has not been evaluated. Hence, this study aims to evaluate the efficacies and recovery times of remimazolam-flumazenil versus propofol in patients undergoing rigid bronchoscopy procedures.

## 2. Materials and Methods

### 2.1. Study Design and Setting

This prospective randomized controlled study was conducted from January 2021 to September 2022 at the Department of Endoscopy of Zhejiang Cancer Hospital, Hangzhou, China. The study was registered at ClinicalTrials.gov (NCT 05468671).

### 2.2. Patient Enrollment and Exclusion Criteria 

Patients who underwent rigid bronchoscopy intratracheal tumor resections or acquired tracheoesophageal fistula stent implantations within the time limit aged > 18 years with an American Society of Anesthesiology (ASA) physical status of II–IV were recruited for the study. Exclusion criteria were as follows: aged < 18 years; refusal to participate; history of schizophrenia, epilepsy, Parkinson′s disease, or myasthenia gravis; severe hepatic dysfunction (Child–Pugh class C); severe renal dysfunction (requiring dialysis); ASA grade of V or above; emergency surgery.

### 2.3. Randomization and Blinding

Patients undergoing endotracheal tumor resections or stent implantations were randomly allocated into either the remimazolam group (Group R) or the propofol group (Group P) in a 1:1 ratio. A randomized allocation sequence was created by an independent research assistant (F.G.) who was not involved in the study using a random number sequence generated from a computer and placed in opaque, concealed, and sequentially numbered envelopes. 

Then, another research assistant (Y.P.) opened the patient envelopes and prepared the appropriate drugs in syringes, infusing tubing and connectors after patients signed the consent forms. The demographic information, vital signs, and recovery times of the patients were recorded on a paper case report form by an attending anesthesiologist (W.Z. or K.X.). Patients and rigid bronchoscopy surgeons were blinded to the group assignment. 

### 2.4. Anesthesia Method Selection

All the patients were continuously monitored with three-lead electrocardiogram, pulse oxygen saturation, and noninvasive blood pressure measurements. Depth of the anesthesia was assessed with a bispectral index (BIS, Covidien IIc, Minneapolis, MN, USA). A TOF-Watch SX (Organon Ireland Ltd, Swords, Ireland) was used to monitor neuromuscular function during surgery to ensure no residual muscle relaxation during anesthesia recovery.

None of the patients used any drugs before induction. After 5 minutes of 100% oxygen preoxygenation, a loading dose of sedative–hypnotic drug was given. The remimazolam general anesthesia group (Group R) received remimazolam (Yichang Renfu Pharmaceuticals Co., Ltd., Yichang, China, lot number: 10T05041) in a 0.4 mg/kg intravenous injection. The propofol general anesthesia control group (Group P) received propofol (Aspen pharmaceuticals Co., Ltd., KwaZulu Natal, South Africa) in a 1.5 mg/kg intravenous injection. Oxycodone (Mundi-pharma, London, UK) at 0.2 mg/kg and rocuronium (MSD, Rahway, NJ, USA) at 0.9 mg/kg were subsequently administered for anesthesia induction. After induction, patients in both groups were ventilated with high-frequency positive-pressure ventilation (HFPPV) (KR-IIJet ventilator, Jiangxi Fifth Machine Tool Factory, Nanchang, China), at respiratory rates of 40–60 breaths/min, I/E ratios of 1:2–1:4, and peak airway pressures of 30 cmH_2_O. The high-frequency ventilation machine was through sputum suction tube or Cook^®^ Airway Exchange Catheter into the rigid bronchoscope, which was in the patient′s airway to achieve ventilation. Ventilation parameters were adjusted to avoid perioperative hypoxemia and hypercapnia and to maintain the end-expiratory carbon dioxide levels at less than 70 mmHg due to permissive hypercapnia.

Group R was administered 1 mg/kg/h of remimazolam [15] and 6–8 μg/kg/h of remifentanil for maintenance. Group P was administered 4–8 mg/kg/h of propofol and 6–8 μg/kg/h of remifentanil for anesthesia maintenance [4]. BIS was maintained at 40–60 in an attempt to maintain sufficient depth of anesthesia. Rocuronium (10 mg) was added according to TOF at the wrist to achieve levels from 1 to 2. Vasoactive drugs were used to maintain mean arterial pressure above 60 mmHg. Warming measures were applied to ensure that patients’ intraoperative body temperatures remained above 36.0 °C.

At the end of the operation, hypnotic agents and remifentanil were discontinued. Then, a laryngeal mask airway (LMA) was inserted into the airway directly after the rigid bronchoscope was removed. Sugammadex (MSD, Hoddesdon, UK) at 2–3 mg/kg was administered as an antagonist of rocuronium in both groups. After TOF > 90%, the specific antagonist of 0.5 mg benzodiazepine flumazenil was administered to reverse remimazolam in group R. The LMA was removed after patients met the extubation criteria.

### 2.5. Operative Process

**Figure 1:**
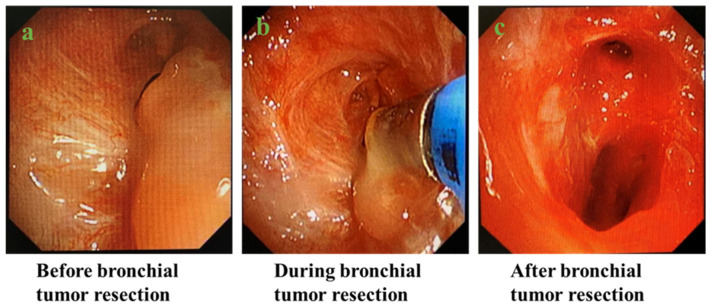
Process of endobronchial tumor resection under rigid bronchoscopy: (**a**) before bronchial tumor resection; (**b**) during bronchial tumor resection; (**c**) after bronchial tumor resection.

Patients with intrabronchial tumors were routinely treated with endotracheal tumor resection under rigid endoscopy (Figure 1).

### 2.6. Data Collection

Demographic information, such as age, gender, weight, past medical history, medications, and the perioperative nutrition status assessment scale (PONS) was recorded upon arrival at the endoscopy department. Patients’ MAP, HR, oxygen saturation, BIS, and TOF were recorded every 5 min until the end of anesthesia.

The primary outcome of this study was the recovery times of the two groups measured in seconds. Using the withdrawal of general anesthesia as the starting point of timing, the end point was established when patients could correctly complete a nod as well as mouth and tongue extension.

The secondary outcomes were time to loss of consciousness (LoC); a modified Brice Questionnaire; the incidence of postoperative delirium (POD); postoperative nausea and vomiting (PONV); the frequency of intraoperative hypotension, bradycardia, stress, or the use of vasoactive drugs.

Hypotension was defined as mean arterial pressure less than 60 mmHg or more than a 20% reduction from baseline. Bradycardia was defined as a heart rate of less than 50 beats per minute or more than a 25% decrease from baseline. Stress was defined as an increase in mean arterial pressure or heart rate more than 20% from baseline.

### 2.7. Statistical Analysis

The sample size was calculated using PASS® version 15.0 (NCSS, LLC, Kaysville, UT, USA). According to our pilot experiment, the mean recovery times of remimazolam-flumazenil and propofol were 174 and 335 s, respectively, whereas the standard deviations were 65 s and 127 s, respectively. Herein, we estimated that a sample size of 12 subjects per group would provide 95% power with an alpha error of 0.05 using a two-sample *t*-test allowing unequal variance. The sample size was increased to 34 subjects (17 per group) to compensate for possible dropouts.

Statistical analyses were conducted using SPSS Windows version 26.0 (IBM, Armonk, NY, USA). Categorical variables were presented as numbers (%). Continuous variables were expressed as mean ± standard deviation for normally distributed variables and median (1Q, 3Q) for non-normally distributed variables. Categorical variables were analyzed using Fisher’s exact test. Continuous variables were analyzed using an unpaired *t*-test or Mann–Whitney U test, as appropriate. Rank variables were analyzed using a 2 independent sample Mann–Whitney U nonparametric test. For repeated measurements of outcomes, a repeated measures ANOVA was used. For all tests, *p* < 0.05 was considered to indicate a statistically significant difference.

### 2.8. Ethics

Approval was sought from the Ethical Committee of Zhejiang Cancer Hospital (No. IRB-2020-406). All procedures performed in the studies involving human participants were in accordance with the ethical standards of the institutional and national research committees. All patients signed written informed consent forms before participating.

## 3. Results

### 3.1. Participants

**Figure 2:**
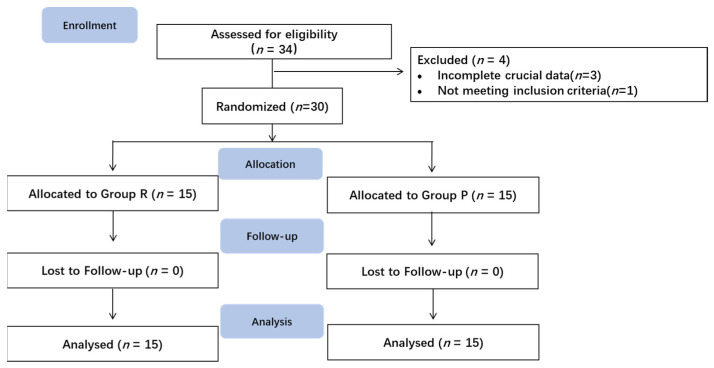
Enrollment diagram.

A total of 34 patients were assessed for eligibility from 4 January 2021, to 8 September 2022 (Figure 2). Thirty patients were included in the data analysis, whereas four patients were excluded. Three patients were excluded due to incomplete recording of key data, and one patient did not meet the inclusion criteria. 

### 3.2. Demographic and Clinical Characteristics

Patient age, sex, BMI, ASA physical status, smoking history, and PONS score did not differ between the two groups (Table 1). The demographic and clinical characteristics of the patients were comparable between the two groups. 

### 3.3. LoC and Recovery Time

**Figure 3:**
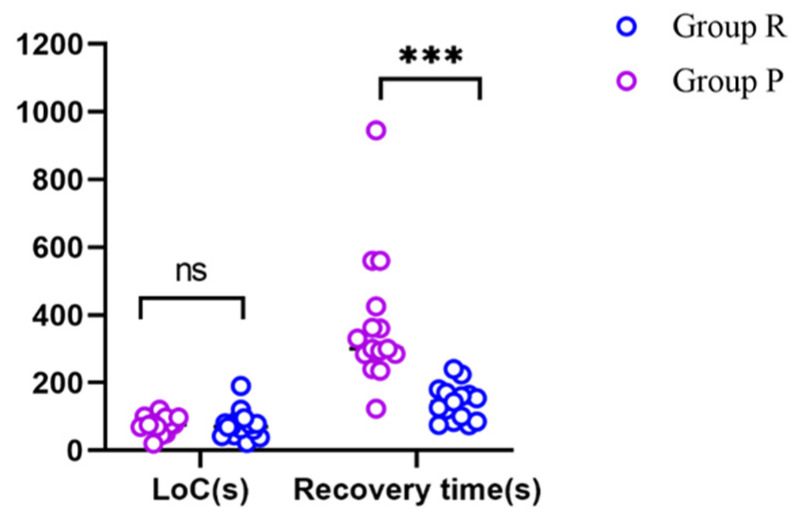
The LoC values and recovery times of the two groups. *** *p* < 0.001; ns: not significantly different; LoC: loss of consciousness.

The time to reach LoC was 76 ± 40 s in Group R and 75 ± 25 s in Group P, and was not significantly different between the two groups. The recovery time was significantly shorter in Group R (140 ± 52 s) than in Group P (374 ± 195 s) (*p* < 0.001) (Figure 3, Table 2).

### 3.4. Parameters during and after Anesthesia

Patient hypotension, bradycardia, average dose of remifentanil, stress times, as well as number of patients using vasoactive drugs did not differ between the two groups. There were no statistically significant differences in the PACU of Observer’s Assessment of Alertness/Sedation (OAA/S) Scale, POD, PONV, modified Brice Questionnaire, or Cough data between the two groups (Table 2).

### 3.5. Hemodynamic Fluctuations

**Figure 4:**
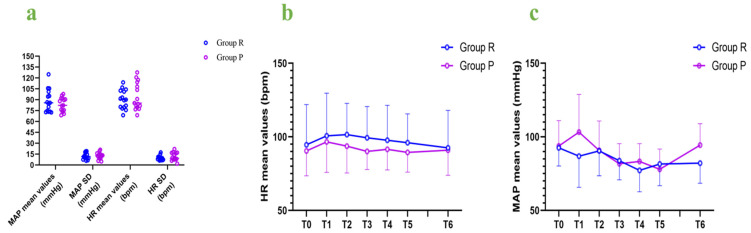
Hemodynamic fluctuations: (**a**) MAP mean values and SD, as well as HR mean values and SD; (**b**) HR mean values at different time points; (**c**) MAP mean values at different time points. MAP: mean arterial pressure; HR: heart rate; SD: standard deviation; bpm: beats per minute; T0: baseline; T1: intubation; T2: 5 min after intubation; T3: 10 min after intubation; T4: 15 min after intubation; T5: 20 min after intubation; T6: extubation.

We calculated the mean and standard deviation of the mean arterial pressure and heart rate for each patient and compared hemodynamic fluctuations between the two groups. The MAP mean values and SD as well as the heart rate mean values and SD were not significantly different between the two groups (Table 2, Figure 4). The MAP mean values and HR mean values were compared at 7 different time points of baseline, intubation, 5 min after intubation, 10 min after intubation, 15 min after intubation, 20 min after intubation, and extubation, and no significant differences were found in the hemodynamic fluctuations between the two groups during the rigid bronchoscopy procedures (Figure 4).

## 4. Discussion

For patients with airway obstruction or tracheoesophageal fistula in advanced cancer, the effect of sedative-hypnotic drugs on respiration cannot be ignored. The purpose of this study was to provide a safer and selective anesthesia method for patients with airway obstruction or tracheoesophageal fistula and at risk of airway obstruction or dysfunction of ventilation at all times. Unlike previous studies, we required flumazenil at the end of the operation, because the operation is not a simple bronchoscopy, but an endotracheal operation that requires a deeper level of anesthesia. Our study showed that the recovery time was significantly shorter for remimazolam-flumazenil than for propofol, which is consistent with previous studies [16]. In the current study, the recovery time was about 140 s, while in another study, the recovery time was approximately 7 min without using flumazenil [17]. However, in other studies, the recovery times have not been shorter for remimazolam than for propofol [14]. This might be explained by the conventional use of the flumazenil antagonist in the present study. The recovery time from anesthesia when using remimazolam-flumazenil was faster than for anesthetization using propofol. We do not recommend the use of antagonists in routine general anesthesia for rapid recovery, rapid recovery can cause panic or discomfort in patients, and the use of antagonists is only an option for emergency difficult airways patients.

Previous studies showed that remimazolam’s onset was 1 to 3 min [4]. The time to reach LoC was about 76 s in Group R and 75 s in Group P, which showed that the onset times between the two anesthetics were similar. The onset time for remimazolam observed here is shorter than in previous studies [18,19]. This might be explained by different induction regimens. In intravenous anesthetics, the time of onset depends directly on the dose. Because the time for anesthetic drugs to reach the maximum effective concentration is constant, increasing the dose can reach the concentration of loss of consciousness earlier [1,20,21]. In addition, the degree and duration of sedation with remimazolam were dose-dependent too [4].

In the present study, the hemodynamic fluctuations did not differ between the two groups. The patients treated with remimazolam needed more vasoactive drugs and remifentanil, but the amounts did not significantly differ between the two groups. It was reported that propofol has an obvious inhibitory effect on the circulatory system and is usually associated with organ injury and poor outcomes [22], whereas remimazolam has the advantage of maintaining circulatory stability, especially for elderly or fatigued patients [6,23,24,25]. Hence, remimazolam is a good choice for patients undergoing rigid bronchoscopy. We used three dimensions (MAP and HR mean values, MAP and HR SD, and seven different MAP and HR time points) to evaluate hemodynamic fluctuations. However, there was no difference in hemodynamics between the two groups in this study. A research reported that hypotension rates during induction were similar between the remimazolam group (0.4 mg/kg) and the propofol group [21]. It was consistent with our findings. Another trial showed that the incidence of hypotension of remimazolam (6 or 12 mg/kg/h) was lower than propofol in induction [14]. It may be related to the higher dose of a single injection, because a higher concentration of remimazolam induced hypotension [21].

There was one patient with POD in the propofol anesthesia group for PACU. However, there were no statistically significant differences in the PACU of Observer′s Assessment of Alertness/Sedation (OAA/S) Scale, POD, PONV, modified Brice Questionnaire, and Cough data between the two groups. It was reported that remimazolam might temporarily impair the quality of recovery [5]; however, this result was not observed here in our study.

Our study has several limitations. First, the small sample size of this study due to the scarcity of cases may affect the results, especially regarding the complications. For example, we did not find differences in hemodynamic fluctuations or adverse events, and this conclusion may change with an increased sample size. Second, arterial blood gas analysis was not conducted in our study. HFPPV is associated with carbon dioxide retention. Although the end-expiratory carbon dioxide was monitored, arterial blood gas analysis is necessary to assess acid–base equilibrium. Third, most patients scheduled for rigid bronchoscopy were breathless and fatigued. Therefore, further study is required to determine whether remimazolam is superior to propofol in patients with ASA physical statuses of II-IV. Fourth, although we controlled the depth of anesthesia between BIS 40 and 60, but we do not record BIS trends in both groups, so the depth of anesthesia is lack of data support. We will collect and analyze BIS data in future studies.

## 5. Conclusions

In conclusion, we evaluated the recovery time from anesthesia using remimazolam-flumazenil versus propofol for rigid bronchoscopy in the current study and found a shorter recovery time for remimazolam-flumazenil than for propofol without the occurrence of dramatic hemodynamic fluctuations or adverse events. Notably, remimazolam-flumazenil allows for faster recovery from anesthesia than propofol, representing a significant advantage in patients with airway obstruction or tracheoesophageal fistula and at risk of airway obstruction or dysfunction of ventilation at all times. We do not recommend the use of antagonists in routine general anesthesia for rapid recovery.

## Figures and Tables

**Table 1:** The demographic and clinical characteristics of the patients.

	Group R (*n* = 15)	Group P (*n* = 15)	*p*-Value
Age	61.13 ± 8.62	60.13 ± 7.24	0.733 ^a^
Sex, male/female	12/3	15/0	0.224 ^b^
BMI	20.08 ± 3.81	21.73 ± 2.89	0.194 ^a^
ASA physical status II/III/IV	7/7/1	11/4/0	0.187 ^c^
Smoking history, *n* (%)	10 (66.7)	9 (60.0)	1.000 ^b^
PONS ≥ 1 *n* (%)	5 (33.3)	2 (13.3)	0.203 ^b^

^a^ Independent *t*-test; ^b^ Fisher’s exact test; ^c^ Mann–Whitney U test. BMI: Body mass index; ASA: American Society of Anesthesiologists; PONS: perioperative nutrition status assessment scale.

**Table 2:** Parameters during and after anesthesia.

	Group R (*n* = 15)	Group P (*n* = 15)	*p*-Value
Loss of consciousness (s)	76 ± 40	75 ± 25	0.952 ^a^
MAP mean value (mmHg)	83.29 ± 9.66	88.79 ± 15.54	0.255 ^a^
MAP SD (mmHg)	12.42 ± 4.48	12.69 ± 4.53	0.869 ^a^
Heart rate mean value (bpm)	94.58 ± 18.89	90.46 ± 13.14	0.825 ^a^
Heart rate SD (bpm)	11.46 ± 5.54	9.57 ± 3.22	0.262 ^a^
Hypotension (*n* (%))	6 (40.0)	5 (33.3)	1.000 ^b^
Bradycardia (*n* (%))	0 (0.0)	1 (6.7)	1.000 ^b^
Stress times	11	13	0.272 ^a^
Average dose of remimazolam or propofol (mg)	59.06 ± 16.89	263.33 ± 100.80	NS
Average dose of remifentanil (ug)	382.73 ± 196.12	365.60 ± 212.95	0.820 ^a^
Number of patients using vasoactive drugs (*n* (%))	6 (40.0)	2 (13.3)	0.215 ^b^
Ephedrine (*n* (%))	1 (6.7)	2 (13.3)	1.000 ^b^
Dopamine (*n* (%))	2 (13.3)	0 (0.0)	0.483 ^b^
Metaraminol (*n* (%))	2 (13.3)	0 (0.0)	0.483 ^b^
Phenylephrine (*n* (%))	1 (6.7)	0 (0.0)	1.000 ^b^
Beta blockers (*n* (%))	2 (13.3)	1 (6.7)	0.550 ^b^
Duration of operation (min)	36.67 ± 19.85	44.40 ± 22.72	0.424 ^a^
Recovery time (s)	140 ± 52	374 ± 195	0.000 ^a^ *
Duration of anesthesia (min)	41.47 ± 19.31	49.60 ± 22.86	0.424 ^a^
Observer’s Assessment of Alertness/Sedation (OAA/S) Scale			
- 3 (*n* (%))	1 (6.7)	1 (6.7)	1.0 ^c^
- 4 (*n* (%))	1 (6.7)	1 (6.7)	
- 5 (*n* (%))	13 (86.7)	13 (86.7)	
POD (*n* (%))	0 (0.0)	1 (6.7)	1.000 ^b^
PONV (*n* (%))	0 (0.0)	0 (0.0)	NS
Modified Brice Questionnaire (*n* (%))	0 (0.0)	0 (0.0)	NS
Cough (*n* (%))	8 (53.3)	8 (53.3)	1.000 ^b^

^a^ Independent *t*-test; ^b^ Fisher’s exact test; ^c^ Mann–Whitney U test; bpm: beats per minute; ^a^ * *p* < 0.001; MAP: mean arterial pressure; SD: standard deviation; bpm: beats per minute; POD: postoperative delirium; PONV: postoperative nausea and vomiting.

## Data Availability

Not applicable.
